# Supplementary material for: Optomechanical vector sensing of new forces at 6 micron separation
Source: Sci Rep. 2026 Jan 13;16:5180. doi: 10.1038/s41598-026-35656-6 (PMC12881458; doi:10.1038/s41598-026-35656-6)
Supplement: Supplementary file 1 — Supplementary Information. [file 41598_2026_35656_MOESM1_ESM.pdf]

Supplementary Material

Summary of measurement configurations

The table below summarizes the measurement configurations used to produce the results in the main text. Each measurement consisted of 10 s of data collected at a sampling rate of 5 kHz. The quoted integration times are not limited by stability of the experiment - rather, the presence of backgrounds mean that there is no marginal benefit to integrating for longer times. Compared to an earlier iteration of the experiment [21], backgrounds and noise have been substantially improved. In addition, two important improvements result in a larger signal: (i) decrease of the $x-$ separation between attractor and microsphere (MS) by $\sim 4\,\mu$m (30%), and (ii) the use of heavier MSs. In the table below, MS diameters are nominal ones, verified using SEM images of ensembles of MSs, while masses are measured in the trap for the MS used. Inferred unequal densities between MSs of different sizes is attributed to differences in the synthesis process.

	**Measurement configuration**					
**MS index**	**MS diameter [μm]**	**MS mass [pg]**	**MS dipole moment [e $\cdot$ μm]**	**MS position [μm]**	**MS dipole moment confinement plane**	**Integration time [10^4 s]**
1	7.56	312	182	(9.6, 1.0, -1.3)	yz	4.5
				(9.6, 1.1, -2.9)	yz	5.4
				(10.3, 1.1, -3.6)	yz	4.5
				(10.2, 1.1, -3.1)	yz	4.95
				(10.3, 1.1, -3.0)	xz	4.95
				(9.2, 1.1, -3.0)	xz	4.95
2	9.98	543	9	(14.7, 0.6, 0.2)	yz	10
3	9.98	1059	48	(11.0, 1.1, -1.5)	yz	4.5
				(11.0, 1.1, -1.6)	yz	4.5
				(13.0, 1.1, -1.6)	yz	4.5

Table S1: Summary of measurement configurations used to produce the results in the main text. The MS position is given with the edge of the attractor defining $x = 0$, and the central gold finger on the attractor defining $y = 0$ and $z = 0$. In all cases, the measurement started with the MS spun up to an angular velocity $\sim 10^6$ rad/s. The ringdown time was separately verified to be long enough that the MS retained angular velocity $\gtrsim 5 \times 10^4$ rad/s at the end of the measurement. Each of the attractor and shield were independently electrically grounded for the duration of all measurements, except rows 4 and 5 for MS 1 (Attractor was DC-biased -50 mV relative to the electrically grounded shield), row 6 for MS 1 (Attractor bias was sinusoidally modulated at 40.5 Hz, 150 $\mathrm{mV_{pp}}$ relative to the electrically grounded shield), and row 2 for MS 3 (Attractor bias was sinusoidally modulated at 46.5 Hz, 200 $\mathrm{mV_{pp}}$ relative to the electrically grounded shield). The quoted values of the dipole moment are obtained for each MS in the trap.
